# The kappa opioid receptor agonist U50,488H did not affect brain-stimulation reward while it elicited conditioned place aversion in mice

**DOI:** 10.1186/s13104-020-05227-7

**Published:** 2020-08-14

**Authors:** Peng Huang, Taylor A. Gentile, John W. Muschamp, Lee-Yuan Liu-Chen

**Affiliations:** Center for Substance Abuse Research (CSAR) & Department of Pharmacology, Lewis Katz School of Medicine at Temple University, 3500 North Broad Street, MERB 851, Philadelphia, PA 19140 USA

**Keywords:** Kappa opioid receptor, U50,488H, Brain-stimulation reward, ICSS, Conditioned place aversion

## Abstract

**Objective:**

Selective kappa opioid receptor (KOR) agonists were shown to produce a dose-dependent depression of brain-stimulation reward (BSR) in the rat intracranial self-stimulation (ICSS) tests. However, limited studies using mice produced less conclusive results. Here the effects of U50,488H were re-examined on BSR in mice with a larger cohort of animals.

**Results:**

Forty C57BL/6J male mice were implanted with the electrodes in medial forebrain bundle. About a week after surgery, mice were subject to ICSS training. Only eighteen passed the two-phase procedures, at which point they readily spun the wheels to obtain reinforcing effect of BSR, and were used for the ICSS tests. Compared with saline (s.c.), U50,488H (2 mg/kg, s.c.) did not have effects on the BSR thresholds within 1 h post-treatment, while it decreased the maximum wheel-spinning rates in a time-dependent manner. In contrast, cocaine (5 mg/kg, s.c.) decreased the BSR thresholds time-dependently without affecting the maximum wheel-spinning rates in the same cohort of mice, demonstrating the validity of our mouse ICSS models. For comparison, U50,488H (2 mg/kg, s.c.) induced significant conditioned place aversion (CPA) in a different cohort of mice without surgeries. Thus, ICSS may not be an appropriate test for KOR agonist-induced aversion in mice.

## Introduction

KOR agonists produce analgesic and anti-pruritic effects, but their development for clinical use has been limited by side effects. KOR agonists cause dysphoric and psychotomimetic effects in humans [1, 2] and aversion in the conditioned place aversion (CPA) test in rats [3, 4] (see [5] for review). More recently, KOR agonists were found to also cause robust CPA in mice and CPA was used to investigate the brain mechanisms underlying the aversive properties of KOR agonists (e.g., [6–8]). In CPA test, animals are conditioned to associate an environmental context with a drug and another context with vehicle in a 2- or 3-chamber apparatus for ≥ 3 sessions and on test day, animals typically avoid the context associated with a KOR agonist. CPA test involves learning and memory, thus alternative tests have been used to allow further mechanistic investigation of KOR agonists-induced aversion.

The intracranial self-stimulation (ICSS) test is a behavioral procedure in which operant responding is maintained by pulses of electrical brain stimulation. ICSS has been used by several research groups to assess the anhedonic properties of KOR agonists including U50,488, U69,593 and salvinorin A, in rats ([9–13], also see [14, 15] for reviews). Nevertheless, the mouse ICSS studies of KOR agonists were limited and produced less conclusive results [8, 16]. In this study, the effects of U50,488H were re-examined in the mouse ICSS test with a larger cohort of animals.

## Main text

### Materials and methods

#### Drugs

U50,488H and cocaine hydrochloride were both provided by Drug Supply Program of National Institute on Drug Abuse (Rockville, MD), dissolved in saline, and injected subcutaneously (s.c.) in a volume of 10 ml/kg body weight.

#### Animals

Male C57BL/6J mice initially weighing 19–22 g (The Jackson Laboratory, Bar Harbor, ME) were given food and water ad libitum on a 12-h light/dark (7 a.m./7 p.m.) cycle. Mice were housed in clear polycarbonate cages (11 × 7 × 5 inches) without enrichment items and with temperatures of 65–75 °F (~ 18–23 °C) and 40–60% humidity. Animals were habituated in the testing room for at least 1 h prior to experimental procedures. Mice were euthanized by CO_2_ asphyxiation followed with cervical dislocation.

#### Other materials

The stainless-steel electrode with both a cathode and an anode for ICSS was purchased from Plastics One via “http://www.invivo1.com” (8IMS3031AIUE, Roanoke, VA). Each cathode was coated with insulation except at the tip, which was cut straight across to a length of about 6–7 mm. Dental resin (4506 and 034522101) was ordered from Den-Mat (Lompoc, CA).

#### Surgery procedure

After 3–7 days of acclimation, mice were anesthetized with ketamine/xylazine (100 mg/kg/10 mg/kg) and the tip (0.25 mm) of a cathode stereotaxically guided to a target near the left medial forebrain bundle (MFB; coordinates in mm from bregma: AP − 1.9; ML + 0.8; DV − 4.8) using the atlas of Franklin and Paxinos [17] and a KOPF stereotaxic apparatus at the level of lateral hypothalamus (LH) as described previously [8, 14, 18]. The electrode was first attached to a stainless-steel screw which served as the electrical ground and was then fixed on the skull with dental cement. Ketoprofen (5 mg/kg) was administered subcutaneously as an analgesic agent during and after surgery. Postoperatively, mice recovered for 1–2 h on a heating pad and were housed individually without enrichment items till the conclusion of the study.

At the end of the experiment, mice were deeply anesthetized with sodium pentobarbital (250–300 mg/kg) and intracardially perfused with 0.9% saline followed by 4% paraformaldehyde. Mouse brains were removed and sectioned (40–50 µm) on a cryostat. Brain sections were stained with cresyl violet and viewed under light microscopy to determine the placement location of the electrode tip.

#### The ICSS test

Our rate-frequency (RF) procedure for ICSS [8, 18] was adapted from protocols described by WA Carlezon, Jr. and EH Chartoff [14] and others [19, 20].

Briefly, after 5–7 days of recovery from surgery, mice were first subjected to 1st-phase training for 60–90 min daily for about a week to determine minimum effective ICSS current (40–130 µA) on a fixed ratio 1 (FR1) schedule. Initially each ¼-turn of the wheel delivered a 0.5 s pulse train of square (0.1 ms × 100 µA) cathodal current at 178 Hz with illumination of the house light (0.5 s). Current was adjusted each day until vigorous, stable responding (~ 3600 responses/hr for > 3 consecutive days with < 20% inter-day variance) was observed.

The 2nd-phase training and the subsequent drug testing sessions, during which the minimum current was held constant for each mouse, consisted of a frequency-dependent 105-min paradigm implemented daily of seven 15-min passes. Within each pass, there were fifteen 1-min trials. For each trial, mice were allowed to respond to one of 15 stimulation frequencies (230–30 Hz) presented sequentially in descending order (0.05 log10 unit steps). Along the 2–3 weeks of the daily 7-pass 2nd phase training, the range of frequencies used was adjusted so that animals responded through the highest 5–8 frequencies stably over latter 6 passes (passes 2 to 7), while pass 1 was a ‘‘warm-up’’ in which nearly all 15 frequencies sustained responding. For each pass, the frequency threshold to maintain BSR responding was computed by least-squares line of best fit analysis using custom-designed software [8, 14]. When mice were observed to show stable mean frequency thresholds of passes 2 to 7 (± 10% over 5 consecutive days), the effects of pharmacological agents would be tested as follows.

For drug testing, each mouse was examined once a day following the above-mentioned 7-pass ICSS paradigm and was repeatedly used for a different treatment (U50,488H and then cocaine) after a wash-out period of more than 48 h. U50,488H, cocaine or saline was injected s.c. at the end of pass 3 (the time point 0 min). For an individual mouse, BSR thresholds or maximum wheel-spinning rates of the four post-treatment passes (passes 4 to 7) were collected at time ranges 0–15, 16–30, 31–45 and 46–60 min. They were normalized to the pre-treatment baselines (the averaged values of thresholds or maximum rates of passes 2 and 3), and presented as “ % baseline BSR threshold” or “ % baseline maximum rate” for each post-treatment pass (at time points of 15, 30, 45 and 60 min).

#### The CPA test

Mouse conditioned place aversion (CPA) test, as a model of dysphoria, was adapted from our method as described previously [8] using an unbiased and counterbalanced two-chamber design. On Day 1, mice were subject to pre-test. On Days 2–4, mice were injected with saline or U50,488H 10 min before each 30-min conditioning session (2 sessions/day) for 3 days. On Day 5 (post-test), the length of time the mouse spent on the U50,488H-paired side was measured. Ten mice were used for each experimental group.

#### Statistical analysis

Data were presented as (mean ± SE) and analyzed with Repeated Measure two-way ANOVA followed by Bonferroni’s Multiple Comparison post hoc tests and Student’s t-tests for ICSS and CPA tests, respectively (Prism 5/GraphPad Software, La Jolla, CA).

### Results

#### Mouse ICSS training

Eighteen of 40 mice implanted with the electrodes passed the two-phase training procedures, at which time they readily spun the wheels to obtain reinforcing effect of BSR. Although their responses during 1st-phase training with a fixed frequency were easily stabilized within 5–7 days of daily training for 36 mice, one half of the mice failed to establish consistent frequency-dependent wheel-spinning responses even after a month of daily 2nd-phase (RF) training. Thus, only 45% (18) of mice post-surgery were proven useful for drug testing. This was largely in accord with our previous experience of mouse ICSS tests [8, 18]. Nevertheless, to the best of our knowledge and surprisingly, it was not mentioned previously in any publications on the mouse ICSS, including reviews [14, 15].

#### U50,488H (2 mg/kg) had no effects on BSR thresholds but decreased maximum rates

**Fig. 1:**
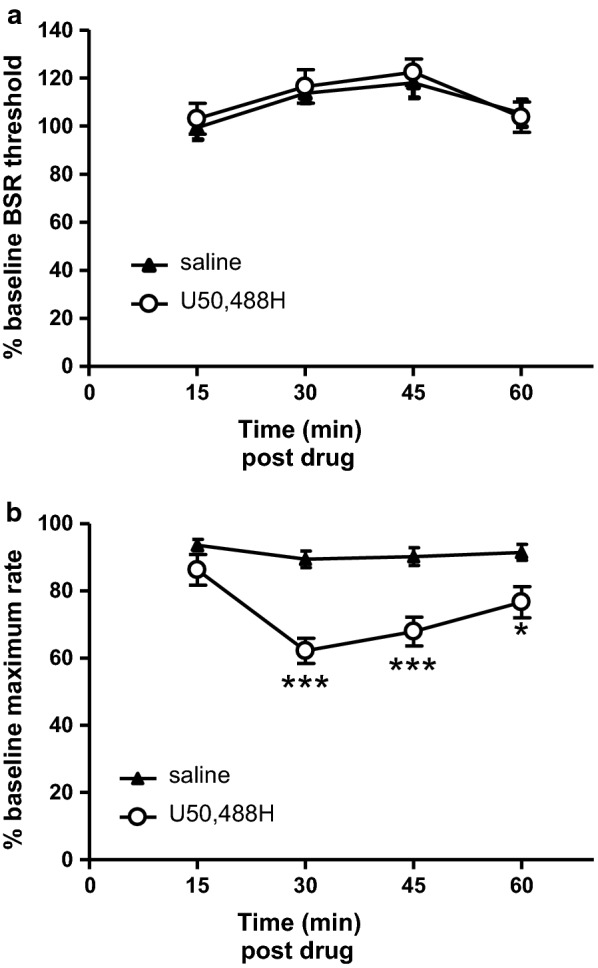
No significant effects of U50,488H were observed on BSR in the ICSS test. Upper panel show data of  % BSR thresholds (**a**) and lower panel demonstrate data of  % maximum wheel-spinning rates (**b**), according to the four post-treatment time intervals (passes) in ICSS tests. The effects of U50,488H at 2 mg/kg were tested on ICSS responding following a seven-pass paradigm as described in the “Materials and methods”. U50,488H or vehicle (saline) was injected subcutaneously at the end of pass 3 (the time point 0 min). BSR thresholds and maximum rates were determined for each pass (time point). The pre-treatment data of passes 2 and 3 (time points − 15 min and 0 min) were averaged and used as “baseline” values for each mouse in every experiment, and the data of passes 4–7 (i.e., post-drug treatment time points 15, 30, 45 and 60 shown here) were normalized to the baseline data accordingly and presented as  % BSR threshold and  % maximum rate. Eighteen mice were used for each experimental group. Data were presented as (mean ± SE) and analyzed by Repeated Measure two-way ANOVA with Bonferroni’s Multiple Comparison post hoc tests (* p < 0.05 and *** p < 0.001)

The eighteen mice were then treated with saline first and then with U50,488H (2 mg/kg, s.c.) on different days. We chose a dose of 2 mg/kg because at this dose U50,488H causes profound CPA ([8] and also see below). The effects of U50,488H on BSR thresholds are shown in Fig. 1a. Repeated Measure 2-way ANOVA analysis revealed that there were no main effects of treatment (U50,488H vs saline, F(1,102) = 0.18) or treatment-time interaction (F(3,102) = 0.15) on BSR thresholds, but there was a main effect of time (F(3,102) = 6.4, p < 0.001) on BSR thresholds. Bonferroni’s post-tests demonstrated no significant difference in BSR thresholds between U50,488H and saline treatments at 15, 30, 45 and 60 min after the treatments. However, U50,488H decreased the maximum rates time-dependently (Fig. 1b). Main effects were observed in treatment (U50,488H vs saline, F(1,102) = 22.5, p < 0.001), time (F(3,102) = 11.6, p < 0.001) and interaction (F(3,102) = 5.75, p < 0.01). U50,488H treatment significantly reduced maximum spinning rates at 30 min (p < 0.001), 45 min (p < 0.001) and 60 min (p < 0.05), but not at 15 min (p > 0.05), after the treatment.

#### Cocaine enhanced BSR

**Fig. 2:**
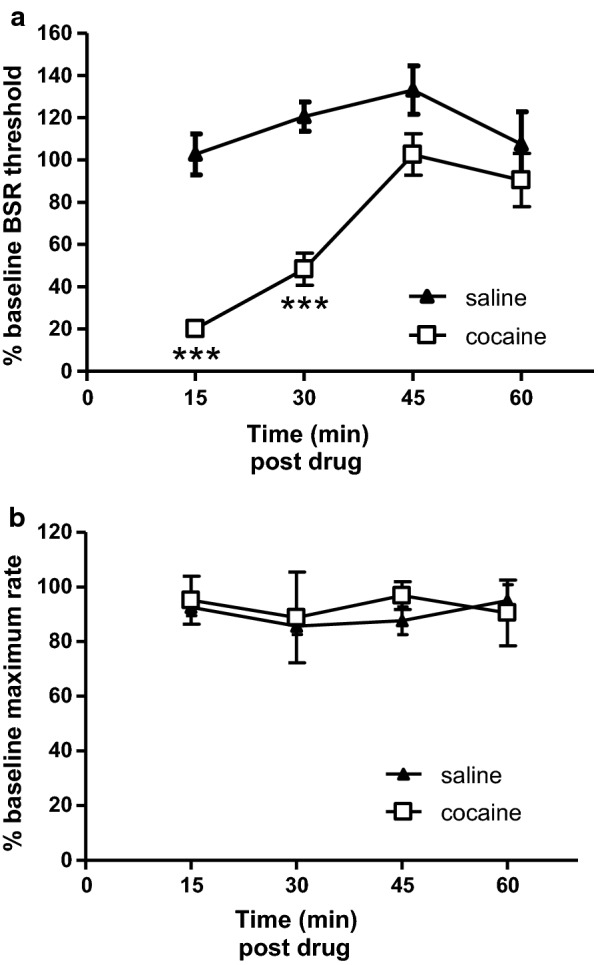
Time (pass)-dependent effects of cocaine on BSR in the ICSS test. Upper and lower panels show data of  % BSR thresholds (**a**) and data of  % maximum wheel-spinning rates (**b**), respectively, according to the four post-drug treatment passes or time points (15, 30, 45 and 60 min). As described in the figure legend of Fig. 1 and following the same seven-pass ICSS paradigm, cocaine (5 mg/kg) or vehicle (saline) was injected subcutaneously at the end of pass 3 (the time point 0 min). The data of post-treatment BSR thresholds and maximum rates data at each of the four time points were normalized to pre-treatment baselines and presented as  % BSR threshold and  % maximum rate, respectively. Six mice were used for each experimental group. Data were presented as (mean ± SE) and analyzed by Repeated Measure two-way ANOVA with Bonferroni’s Multiple Comparison post hoc tests (*** p < 0.001)

To ascertain the validity of ICSS test in these mice, we tested effects of cocaine in these mice. More than 48 h after U50,488H treatment, six mice were treated with saline and then cocaine (5 mg/kg, s.c.) on different days and ICSS responding was recorded (Fig. 2). Repeated Measure 2-way ANOVA demonstrated main effects of treatment (cocaine vs saline, F(1,30) = 36.3, p < 0.001), time (F(3,30) = 12.7, p < 0.001) and interaction (F(3,30) = 5.62, p < 0.01) on BSR thresholds. Bonferroni’s post-tests revealed that compared with saline, cocaine treatment significantly lowered BSR thresholds at 15 min (p < 0.001) and 30 min (p < 0.001), but not at 45 min and 60 min (p > 0.05), post treatments (Fig. 2a). In contrast, in terms of maximum rates (Fig. 2b), there were no main effects in treatment (cocaine vs saline, F(1,30) = 0.099), time (F(3,30) = 0.32) and interaction (F(3,30) = 0.29).

#### U50,488H produced conditioned place aversion

**Fig. 3:**
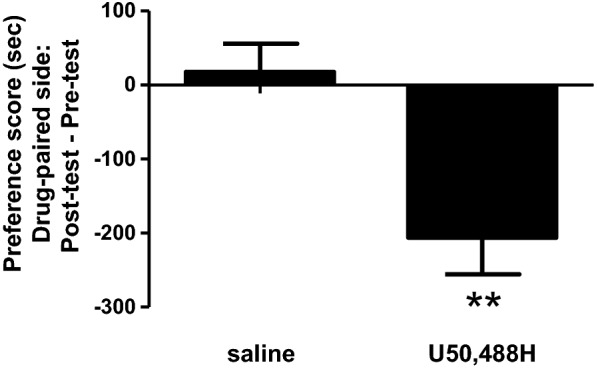
The effects of U50,488H in the CPA test. Briefly, as described in the “Materials and methods”, mice underwent pre-tests on Day one and then conditioned twice a day (one saline and one U50,488H sessions) for 3 days and tested on Day 5. The data shows the time the mouse spent during the post-test subtracting the amount of time spent during the pre-test. Ten mice were used for each experimental group. Data were presented as (mean ± SE) and analyzed by Student’s t-tests (** p < 0.01)

In a separate cohort of male C57BL/6J mice without surgeries, U50,488H (2 mg/kg, s.c.) induced significant aversion (p < 0.01, Student’s *t* test), compared to saline (Fig. 3).

### Discussion

In the current study, U50,488H at 2 mg/kg vs saline did not change BSR thresholds at all 4 time points within 1 h post treatment while it attenuated the maximum rates time-dependently in the mouse ICSS test. The observations are different from our previous findings [8], in which U50,488H (s.c.) at 0.5, 1.0 and 2.5 mg/kg increased the BSR thresholds to ~ 1.2–1.3-fold level of saline without changing the maximum rates [8].

The previous  % BSR threshold data were shown as the average of those at two time points (30 min, 45 min) [8]. When the current data were presented the same way, no difference was found between saline- and U50,488H-treated groups of mice (115.9 ± 3.5 vs 119.5 ± 4.8, p = 0.54, *Student’s t*-*test*). On the other hand, we showed that compared with saline, cocaine enhanced the BSR dramatically, reminiscent of several previous findings using C57BL/6 J mice [21–23], demonstrating the validity of our mouse ICSS models.

It should be noted that, in the previous study [8], 2.5 mg/kg of U50,488H showed a trend of decreasing maximal wheel-spinning rates, but did not reach statistical significance. In the current study, with more animals used for each treatment group (N = 18 vs 12 [8]), we observed that 2 mg/kg of U50, 488H decreased the maximum rates in a time-dependent manner with peaked effects at 30 min post treatment, which was in accord with our data from mouse rotarod tests [8] and locomotor tests (unpublished). It should be emphasized that, in the current study, 2 mg/kg of U50,488H failed to affect BSR thresholds even when the wheel-spinning rates were decreased at 30, 45, and 60 min time points, which was likely due to poorer motor coordination induced by U50,488H.

Roth and colleagues used a similar ICSS paradigm to evaluate two other selective KOR agonists in male C57BL/6 mice [16]. They found that U69,593 and salvinorin A increased BSR threshold in time- and dose-dependent manners. However, at time points and doses that the drugs increased BSR thresholds (U69,593 0.3 mg/kg at 15 and 30 min, 1 mg/kg at 15, 30, 45 and 60 min post treatment; salvinorin A 1 mg/kg at 30 min post treatment), they also decreased the maximum wheel-spinning rates, thus confounding data interpretation. Moreover, U69,593 0.3 mg/kg at 45 min decreased the maximum rate significantly, but still failed to affect the BSR threshold, implying that if there was any aversive effect induced by U69,593, the effect was too little to be detected by the ICSS test in mice. One exception is 1 mg/kg salvinorin A at 15 min, in which it increased BSR threshold without affecting maximal spinning rate, demonstrating the anhedonia unambiguously [16]. In contrast, both U69,593 and salvinorin A at 1 mg/kg produced robust CPA in mice in the same study [16]. In fact, Brust et al. [11] reported similar findings in rats that U50,488H increased BSR threshold at doses that decreased the maximal spinning rates.

### Conclusion

The current and previous results from our lab revealed that using the mouse ICSS test to assess anhedonic effects of U50,488H has yielded inconsistent results. The results from Dr. Roth’s group [16] showed an important confounding factor in the mouse ICSS test that KOR agonists inhibited mouse motor activities, which impacts on wheel-spining rates and renders the data interpretation equivocal. In summary, mouse data from both of our group and Dr. Roth’s group demonstrated that (1) in many cases, KOR agonists increased BSR thresholds and decreased maximum rates simultaneously and (2) KOR agonists, in some cases, had no effects on BSR thresholds even when they decreased maximum rates. Thus, lower doses of KOR agonists were required to be used in order to get their “clean” effects on BSR thresholds without being confounded by their inhibitory effects on wheel spinning rates. However, lower doses of drugs yielded lower level of effects on BSR thresholds, which turned to be too subtle to be reproduced consistently.

On the contrary, KOR agonists have produced consistent and robust aversion in the CPA test in mice, in which their acute hypolocomotion does not affect the behavioral endpoint.

## Limitations

KOR agonists did not consistently enhance BSR thresholds in mice. When enhancements were observed under certain (time, dose) conditions, in most cases KOR agonists also reduced maximum spinning-rate, thus confounding data interpretation.

## Data Availability

Not applicable

## Abbreviations

BSR: Brain-stimulation reward
CPA: Conditioned place aversion
ICSS: Intracranial self-stimulation
KOR: Kappa opioid receptor
LH: Lateral hypothalamus
MFB: Medial forebrain bundle
RF: Rate-frequency
s.c.: Subcutaneously
U50,488H: (±)-(trans)-3,4-dichloro-*N*-methyl-*N*-[2-(1-pyrrolidiny)-cyclohexyl]-benzeneacetamide methanesulfonate salt
U69,593: (+)-(5α,7α,8β)-*N*-Methyl-*N*-[7-(1-pyrrolidinyl)-1-oxaspiro[4.5]dec-8-yl]-benzeneacetamide
